# Nanocomposites used in the treatment of skin lesions: a scoping review

**DOI:** 10.1590/1980-220X-REEUSP-2023-0338en

**Published:** 2024-05-13

**Authors:** Wevernilson Francisco de Deus, Camylla Layanny Soares Lima, Ana Luiza Barbosa Negreiros, Phellype Kayyaã da Luz, Raylane da Silva Machado, Grazielle Roberta Freitas da Silva

**Affiliations:** 1 Universidade Federal do Piauí, Programa de Pós Graduação em Enfermagem, Teresina, PI, Brazil.; 2 Universidade Federal do Piauí, Teresina, PI, Brazil.; 3 Colégio Técnico de Bom Jesus, Bom Jesus, PI, Brazil.; 4 Colégio Técnico de Floriano, Floriano, PI, Brazil.

**Keywords:** Wounds and Injuries, Skin Ulcer, Nanocomposites, Nanogels, Heridas y lesiones, Úlcera cutánea, Nanocompuestos, Nanogeles, Ferimentos e Lesões, Úlcera Cutânea, Nanocompostos, Nanogéis

## Abstract

**Objective:**

To map the nanocomposites used in the treatment of skin lesions.

**Method:**

A scoping review, according to the Joanna Briggs Institute methodology, carried out on eight databases, a list of references and Google Scholar to answer the question: “Which nanocomposites are used as a cover for the treatment of skin lesions?”. Two independent reviewers selected the final sample using inclusion/exclusion criteria using the EndNote^®^ and Rayyan programs. Data was extracted using an adapted form and reported using the PRISMA checklist extension, and the protocol was registered in the Open Science Framework (OSF).

**Results:**

21 articles were selected, with nanofibers, nanogels and nanomembranes as the nanocomposites described in wound healing, alone or in association with other therapies: negative pressure and elastic. Silver nanomaterials stand out in accelerating healing due to their antimicrobial and anti-inflammatory action, but caution should be exercised due to the risk of cytotoxicity and microbial resistance.

**Conclusion:**

Nanocomposites used in wound treatment are effective in accelerating healing and reducing costs, and the addition of bioactives to nanomaterials has added extra properties that contribute to healing.

## RESUMO

**Objetivo:**

Mapear os nanocompostos utilizados no tratamento de lesões cutâneas.

**Método:**

Revisão de escopo, conforme metodologia Joanna Briggs Institute, realizada em oito bases de dados, lista de referências e Google Scholar para responder à pergunta: “Quais os nanocompostos utilizados como cobertura para o tratamento de lesões cutâneas?”. Dois revisores independentes, selecionaram a amostra final mediante critérios de inclusão/exclusão usando os programas EndNote^®^ e Rayyan. Os dados foram extraídos com formulário adaptado e reportados pela extensão do checklist PRISMA, o protocolo foi registrado na *Open Science Framework* (OSF).

**Resultados:**

21 artigos selecionados, trouxeram nanofibras, nanogéis e nanomembranas como os nanocompostos descritos na cicatrização de feridas, isolados ou em associação a outras terapias: pressão negativa e elástica. Os nanomateriais com prata destacam-se em acelerar a cicatrização pela ação antimicrobiana e anti-inflamatória, recomenda-se cautela no uso pelo risco de citotoxicidade e resistência microbiana.

**Conclusão:**

Os nanocompostos utilizados no tratamento de feridas são eficientes em acelerar a cicatrização e reduzir custos, a adição de bioativos aos nanomateriais agregaram propriedades extras que contribuem com a cicatrização.

## RESUMEN

**Objetivo:**

Mapear los nanocompuestos utilizados en el tratamiento de lesiones cutáneas.

**Método:**

Revisión de alcance, según la metodología del Instituto Joanna Briggs, realizada sobre ocho bases de datos, una lista de referencias y Google Scholar para responder a la pregunta: “¿Qué nanocompuestos se utilizan como cobertura para el tratamiento de lesiones cutáneas?”. Dos revisores independientes seleccionaron la muestra final mediante criterios de inclusión/exclusión utilizando los programas EndNote^®^ y Rayyan. Los datos se extrajeron mediante un formulario adaptado y se notificaron utilizando la extensión de la lista de comprobación PRISMA, y el protocolo se registró en el Open Science Framework (OSF).

**Resultados:**

Se seleccionaron 21 artículos, con nanofibras, nanogeles y nanomembranas como los nanocompuestos descritos en la cicatrización de heridas, solos o en asociación con otras terapias: presión negativa y elástica. Los nanomateriales con plata destacan en la aceleración de la cicatrización por su acción antimicrobiana y antiinflamatoria, pero se recomienda precaución en su uso por el riesgo de citotoxicidad y resistencia microbiana.

**Conclusión:**

Los nanocompuestos utilizados en el tratamiento de heridas son eficaces para acelerar la cicatrización y reducir costes, y la adición de bioactivos a los nanomateriales ha añadido propiedades adicionales que contribuyen a la cicatrización.

## INTRODUCTION

Nanotechnology has had a major impact on the development of science and specifically on technological innovation. The synthesis and design of nanoscale structures present the most diverse possibilities for use in health care and scientific research ^(1,2)^. In this context, there is the manufacture of nanocomposites, defined as structures in which at least one of their components is on the nanometric scale (1 to 1000 nanometers)^(3,4)^.

With its ability to modulate chemical properties, nanotechnology is an efficient strategy for advanced wound care. It offers a wide variety of nanomaterials for topical use in isolation, or in conjunction with scientific consensus therapies, in specific skin lesions, promoting a marked improvement in healing in both scenarios^(5)^.

Among the different designs presented, it is worth highlighting: scaffolds with three-dimensional and porous structures (scaffolds); fibrous structures interwoven with polymeric filaments and a large surface area (nanofibers); three-dimensional polymer networks containing hydrophilic and cross-linked groups (nanogels); interphases with a selective or semi-permeable barrier through the combination of organic and inorganic compounds (nanomembranes); hollow cylinders or tubes (nanotubes). In this way, nanocomposites have various possible uses, such as: controlled release or transportation of drugs and bioactive substances, support for cell growth and differentiation, bone and tissue regeneration^(6-8)^.

In this scenario, research involving the transport of bioactive substances between the blood-brain barrier and mimetics of the extracellular matrix stands out^(2)^. This ability to mimic the extracellular environment and provide differentiated cell growth makes nanomaterials a great promise for the tissue regeneration process^(6)^. The manufacture of nanocomposites with biocompatible materials, providing mechanical support without a biological response in the host organism, gives them the ability to modulate the complex healing process and accelerate tissue repair^(7,9)^.

Depending on the progression of the healing process, skin lesions can be classified as acute or chronic. In acute lesions, the hemostasis process is triggered after vascular rupture with a continuous and dynamic evolution of the healing phases, the dominant physiological changes are vascular and exudative, located at the point of aggression with retraction of the margins in up to three weeks; in chronic lesions, there is a staging or sequential deviation of the healing phases, the inflammatory phase remains for a long time, compromising an orderly repair and prolonging the retraction of the margins for a period of more than three weeks^(10)^. Tissue repair still represents a major clinical and scientific challenge, in which specialized efforts are directed at reducing the physiological, functional, institutional and financial impact of a wound^(10,11)^.

This challenge is driving several researchers towards the possibility of using innovative materials in scientific research that can speed up the wound healing process^(10,11)^. Thus, some properties, such as biocompatibility characteristics, designs of structures similar to the extracellular matrix and the carrying of bioactive substances; added to the promising results in the area of skin care resulting from the efficiency of nanocomposites in preventing skin lesions^(12)^ give nanomaterials too much scientific interest. However, knowledge of nanotechnology, the nanomaterials that can be made, their possible applications and results is prevalent among professionals in the field of biomedical and materials engineering^(11)^.

The development of research using nanotechnology is among the thematic priorities for the period from 2020 to 2023 within the scope of Brazil’s Ministry of Science, Technology, Innovation and Communications. Ministerial Ordinance No. 1122 of March 19, 2020 reinforces that nanotechnology can contribute to the innovation base for products that are intensive in scientific and technological knowledge^(13)^.

From this perspective, professionals directly involved in health care, especially nursing in the context of tissue injury care, need to have ownership and mastery of technological innovation, including the use of nanocomposites in the healing process.

However, there is a notorious conceptual and knowledge gap at national and international level regarding the specification and possibilities of using nanomaterials as a therapeutic covering in the healing process. A preliminary search was conducted in July 2022 in the Virtual Health Library and in the databases COCHRANE, CINAHL, EMBASE, SCOPUS, Web of Science and MEDLINE via PubMed, where until July 15, 2022 no scoping reviews or systematic reviews in progress or completed were found that addressed aspects related to the topic of interest.

Therefore, the relevance of this scoping review proposal is justified, which aims to map nanocomposites used as coverings in skin lesions during the healing process. It is hoped that these materials can be used in future research in order to contribute to professional assistance.

## METHOD

This is a scoping review of the literature, developed according to the methodology proposed by the Joanna Briggs Institute (JBI)^(14)^. The findings of this review were reported according to the PRISMA 2020 checklist (Preferred Reporting Items for Systematic reviews and Meta-Analyses extension^(15)^. The research protocol for this study is registered on the Open Science Framework (OSF) platform (https://osf.io/2gudk/).

### Research Question

To formulate the guiding question, the acronym PCC was used, in which “P” represents the population (people with skin lesions); “C” the concept (nanocomposites or nanogels); and “C” the context (broad, without restriction). Thus, the guiding question of this study was: “Which nanocomposites are used as a cover for the treatment of skin lesions?”.

### Sources of Information and Inclusion Criteria

We considered studies published in full, with no restrictions on methodological design, languages or time limits. We considered articles published in journals and publications from the gray literature, such as course completion papers, theses and dissertations.

Inclusion/exclusion criteria were defined for each letter of the acronym PCC. Thus, studies whose population was patients with skin lesions were included. Regardless of the etiology, whether acute wounds or chronic wounds, patients with pre-existing illnesses in home care, outpatient care or in a healthcare institution were considered. Within the concept, the studies included used the nanocomposites or nanogels developed as the primary covering for wounds, regardless of the synthesis technique or design. Studies in which the coverings were applied exclusively to assess antimicrobial action, without evaluating healing progress, were disregarded. The context of this review was broad, with no restrictions on the context of care (hospital, home or outpatient) or any specific area of knowledge.

### Search Strategy

Searches were carried out in the following databases: Medical Literature and Retrieval System online (MEDLINE) via National Center for Biotechnology Information (NCBI/PubMed), Latin American and Caribbean Literature in Health Sciences (LILACS), Nursing Database (BDENF) and Spanish Bibliographic Index in Health Sciences (IBECS), via the Virtual Health Library, EMBASE via Elsevier, COCHRANE, CINAHL and Web of Science (WOS) were accessed via the Journal Portal of the Coordination for the Improvement of Higher Education Personnel (CAPES). Additional strategies included searching Google Scholar and cross-referencing. The searches were conducted between July and December 2022 and updated in February 2024.

The databases were searched using controlled descriptors from the Health Sciences Descriptor Database (DeCS), Medical Subject Headings (MeSH), Emtree and CINAHL titles, as well as keywords and synonyms. In order to broaden the findings, the strategies were defined by the reviewers with the help of a librarian. Chart 1 shows the construction syntax, descriptors/keywords and Boolean operators used in the high-sensitivity search in the MEDLINE/ NCBI/PubMed database. The other strategies can be found in the scoping review protocol: (https://osf.io/2gudk/).

**Chart 1:** Construction syntax, descriptors/keywords and Boolean operators used in the MEDLINE/NCBI/PubMed database – Teresina, PI, Brazil, 2024.

Database	Search Strategy
MEDLINE/PubMed N = 1.754	(“Wounds and Injuries”[Mesh Terms] OR “Skin Ulcer”[Mesh Terms] OR (Wounds and Injuries) OR (Injuries and Wounds) OR (Wounds and Injury) OR (Injury and Wounds) OR (Wounds, Injury) OR (Injuries, Wounds) OR (Injuries) OR (Injury) OR (Wounds) OR (Wound) OR (Skin Ulcers) OR (Ulcer, Skin) OR (Ulcers, Skin) OR (Dressing)) AND (“Nanocomposites”[Mesh Terms] OR ” Nanogels”[Mesh Terms] OR (Nanocomposite Gels) OR (Nanocomposite Gel) OR (Gel, Nanocomposite) OR (Nanocomposite Hydrogels) OR (Nanocomposite Hydrogel) OR (Hydrogel, Nanocomposite) OR (nanofiber) OR (Scaffolds AND Nanocomposites)) Filters: Humans

Source: Authors.

### Selection of Studies

After searching the databases, the results found were uploaded to EndNote web (Clarivate Analytics, Pennsylvania, United States of America) where duplicates were identified and removed. The Rayyan software (Qatar Computing Research Institute, Doha, Qatar) was used to analyze, select and exclude the articles, where the remaining duplicates were also analyzed and excluded.

Screening and evaluation of the references found was carried out by two reviewers in a blind evaluation, and divergent cases were evaluated by a third reviewer. The pre-selected studies were read in full and assessed against the inclusion criteria already defined.

### Data Extraction

A tool developed by the reviewers was used to extract data from the included articles, which was based on the model available in the JBI manual and is available for consultation on the OSF platform (https://osf.io/2gudk/).

### Presentation of Results

The data extracted was presented in the form of tables and narrative discussion, taking into account the aim of this scoping review.

## RESULTS

After selecting the databases using the search strategies set up, 5,614 articles were retrieved: MEDLINE/PubMed N = 1,754, LILACS N = 19, BDENF N = 5, IBECS N = 2, EMBASE N = 1,203, COCHRANE N = 62, WEB OF SCIENCE N = 758, CINAHL N = 1,811, grey literature N = 100, list of references N = 14. Duplicates were then excluded and the title and abstract were read, applying the inclusion/exclusion criteria.

Of those eligible for full reading, 106 articles were removed according to the exclusion criteria: 48 were animal models, 18 in vitro studies, 3 non-cutaneous lesions, 3 studies the material was not nanocomposite, 13 referred to nanotechnology as a potential perspective for healing, 2 discussed electrospinning, 5 did not deal with the application of the nanocomposite, 9 the objective was antimicrobial potential, 3 did not specify the population and 1 the object of study was absorption rate of the dressing and 1 was discarded because it had undergone a retraction. In this review, the final sample totaled 21 selected studies.

**Figure 1:**
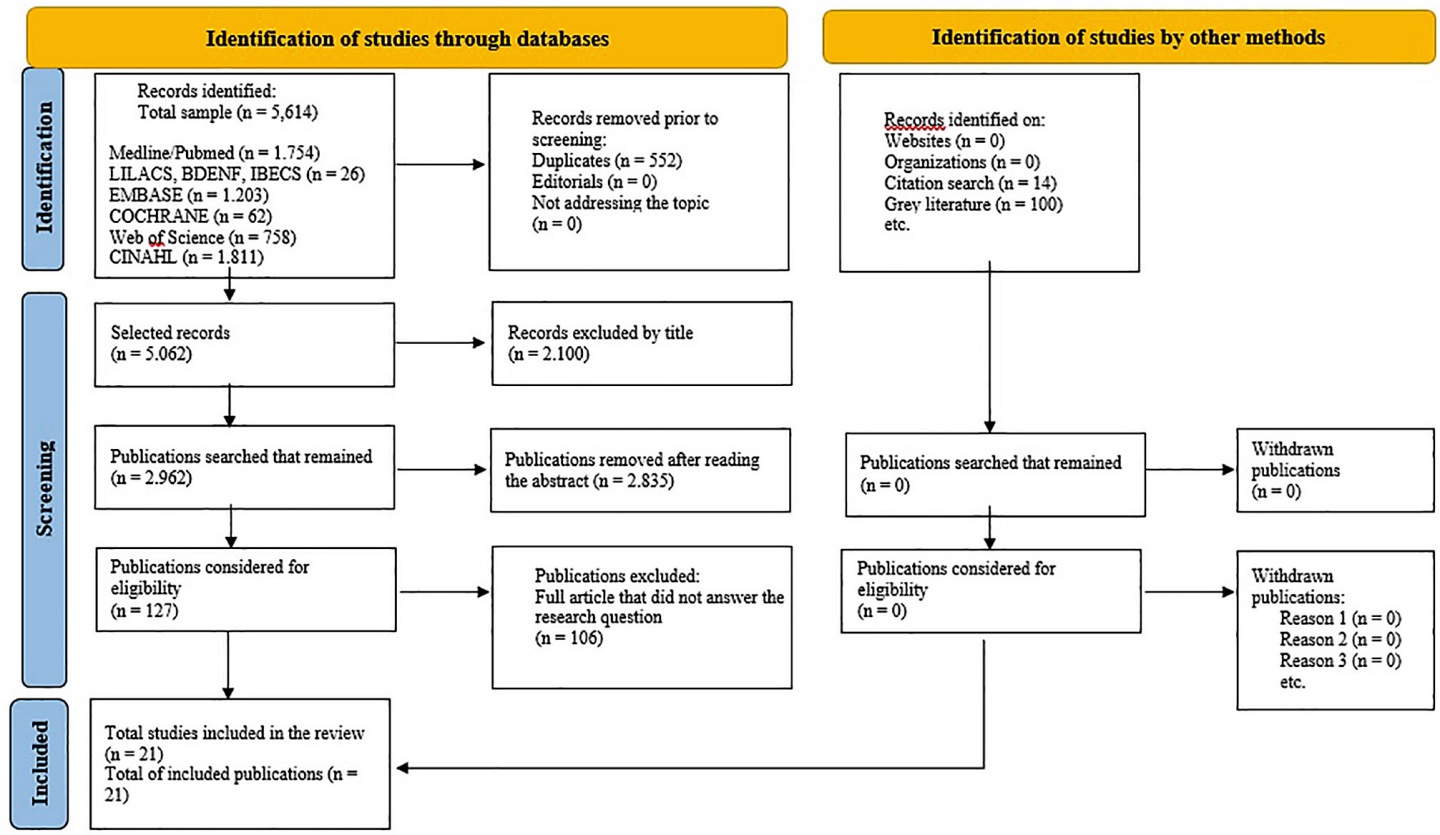
PRISMA flowchart for the selection of review articles. Teresina, PI, Brazil, 2024. Source: Prepared by authors based on PRISMA 2020^(15)^.

The process of searching for and selecting the studies in this review is shown in the flowchart (Figure 1), according to the recommendations of the JBI, following a checklist adapted from PRISMA.

Among the 21 studies included in this review, the publication years were: 2021 with 5 (23.8%), followed by 4 (19%) in 2019, 3 (14.2%) in 2023, 2 (9.5%) in 2012, 2 (9.5%) in 2016 and 2 (9.5%) in 2018 and one (4.7%) in 2015, one (4.7%) in 2017 and finally one (4.7%) in 2022. The English language was unanimous (100%).

The geographical distribution of publications was concentrated in the United States (USA) and China with 3 (14.2%), 2 (9.5%) in the Czech Republic, Egypt and Iran and one (4.7%) in Greece, France, Canada, Brazil, Malaysia, Sweden, Poland, Mexico and Switzerland. As for the professional areas responsible for the research, nursing was only responsible for 2 (9.5%) publications independently and 3 (14.2%) jointly with medicine, with a predominance of articles in the area of medicine (dermatology) with 6 (28.5%), oncology and traumatology with one (4.7%) each, pharmacology independently 2 (9.5%) and in partnership with medicine there were also three (14.2%) and finally 3 (14.2%) publications from medicine with nanoscience (materials).

In terms of methodological design, there were five clinical trials^(16–20)^, three case studies^(21–23)^, eight randomized clinical trials^(24–31)^, one retrospective^(32)^ and prospective^(33)^ study each and three case series^(34–36)^ with similar study objects on the use of various nanocomposites in the treatment of skin lesions. The etiologies of the wounds were: burns^(24)^, radiodermatitis^(16)^, toxic necrolysis epidermitis^(21)^ and surgical necrolysis^(31)^ with one study each, pressure injuries^(20,29)^ with two studies, venous ulcers with six studies^(17,19,22,25,30,35)^ and diabetic foot ulcers with nine studies^(18,23,26–28,32–34,36)^.

Among the articles selected, there was a wide variety in the design of the nanocomposites tested, which included nanofibers^(16–20,27–30)^, nanogels^(23,26,33)^, nanoemulsion^(31)^, nanomembrane^(35)^, synthetic dressings with at least one of the components on a nanoscale^(21,24,25,32,36)^, sprays with nanoparticles^(34)^, and nanocapsules^(22)^ in compression stockings with a predominance of studies using nanosilver^(21,24,27,32–34)^ with good results and some recommendations regarding its use (Chart 2).

**Chart 2:** Data extracted from the studies included in the scoping review – Teresina, PI, Brazil, 2024.

	Journal/field	Year/country	Title	Type of nanocomposite	Nanocomposite composition	Etiology of injuries	Outcome	Study type
1	MDPI Medicine(Oncology)	2021 Greece	Management of Acute Radiodermatitis in Non-Melanoma Skin Cancer Patients Using Electrospun Nanofibrous Patches Loaded with Pinus Halepensis Bark Extract^(16)^	Nanofiber Commercial: NA*	1*- Polyethylene oxide and cellulose acetate 2*- Aqueous extract of Pinus halepensis bark (PHBE)	Acute Radiodermatitis	In contrast to the reference product, the PHBE patch showed anti-inflammatory activity and restored most skin parameters to normal levels 1 month after use, contributing to the prophylaxis and successful management of acute radiodermatitis. Beneficial effects were observed on the RTOG scale, TEWL, erythema, hemoglobin concentration, skin texture and subjective experience of itching and pain, while no statistically significant variation was observed between the two interventions for hydration and melanin.	Clinical trial
2	Evidence-Based Complementary and Alternative Medicine HINDAWI Nursing	2017 China	A Pilot Randomized, Controlled Study of Nanocrystalline Silver, Manuka Honey, and Conventional Dressing in Healing Diabetic Foot Ulcer^(27)^	Nanofiber Commercial: NA	1- Polyethylene 2- Nanocrystalline silver	Diabetic foot ulcer (DFU)	The proportions of complete healing were 81.8%, 50% and 40% in the nanocrystalline silver (nAg), manuka honey (MH) and conventional (paraffin tulle) groups, respectively. The rate of size reduction was potentially higher in the nAg group (97.45%) than in the MH group (86.21%) and the conventional group (75.17%).	Open randomized clinical trial
3	Acta Chir Orthop Traumatol Czech Medicine (Orthopedic and Traumatology)	2022 Czech Republic	Management of Leg Ulcers Using Combined PRP Therapy on a Nanofiber Carrier: Results of a Pilot Study^(17)^	Nanofiber Commercial: NA	1- Polycaprolactone (PLC) 2- Platelet-rich plasma (PRP)	Leg ulcers	When comparing healing progress between day 0 and day 168, there was a statistically significant decrease in surface area in both groups. As for depth, there was a statistical difference, and the experimental group (PLC/PRP) had a better evolution compared to the control group (Mepilex^®^). The experimental group also had more healed ulcers.	Clinical Trial (Pilot Study)
4	Acta Medica Mediterranea Medicine and Nursing	2023 China	Clinical effect and collaborative nursing of polycaprolactone/gelatin nanofiber membrane in the treatment of stage 2 pressure injury^(20)^	Nanofiber Commercial: NA	1-Polycaprolactone (PCL) 2- Collagen	Stage 2 pressure injury	The lesions were measured using the PUSH scale and there was a reduction in the area from 19.48 ± 15.41 on admission to 4.26 ± 3.47 in the third week (P > 0.05) and after 28 days of the nursing intervention, all the dimensions of the quality-of-life scores in both groups improved, with the scores of the experimental group higher than those of the control group (P > 0.05).	Clinical trial
5	International Immunopharmacology Medicine (Immunology, Endocrinology), Pharmacology, Biology.	2021 Iran	Improved wound healing of diabetic foot ulcers using human placenta-derived mesenchymal stem cells in gelatin electrospun nanofibrous scaffolds plus a platelet-rich plasma gel: A randomized clinical trial^(28)^	Nanofiber Commercial: NA	1- Gelatin 2- Mesenchymal stem cells (hPDMSCs) and platelet-rich plasma (PRP)	Diabetic foot ulcers	The reduction in wound size was 66% in group A (nanofiber with stem cells), 71% in group B (nanofiber with stem cells + PRP) and 36% in control group C (conventional therapy). A significant difference in wound closure and no pain was observed between groups A and B compared to control group C (p < 0.05), but there was no difference between groups A and B. Implantation of hPDMSCs in PRP accelerated wound healing and improved clinical parameters in DFU patients.	Randomized clinical trial
6	Pharmacological Reports Pharmacology	2021 Poland	Alleviating neuropathy of diabetic foot ulcer by co-delivery of venlafaxine and matrix metalloproteinase drug-loaded cellulose nanofiber sheets: production, in vitro characterization and clinical trial^(18)^	Nanofiber Commercial: NA	1- Cellulose 2- Venlafaxine (VEN) and Doxycycline (DOX)	Diabetic foot ulcers (DFU)	Ulcer size showed a faster reduction after 12 weeks in the treatment group (12.22 ± 6.53 cm^2^ to 5.00 ± 3.46 cm^2^) compared to the control group (13.1 ± 5.05 cm^2^ to (7.06 ± 4.55 cm^2^). The distance walked without pain increased in the treated group (34.00 ± 6.86 m to 263.50 ± 59.63 m) while the control increased from (35.5 ± 3.27 m to 105.5 ± 17.01m) (p < 0.001). Microscopic studies of the skin showed the formation of new capillary beds. The nanofiber accelerates healing and reduces neuropathy in the DFU of diabetic patients.	Clinical trial
7	Experimental Dermatology Medicine (Dermatology)	2012 Czech Republic	Light-activated nanofibre textiles exert antibacterial effects in the setting of chronic wound healing^(19)^	Nanofiber Commercial: NA	1- Polyurethane (NT) 2- Tetraphenylporphyrin (TPP) photosensitizer	Leg ulcers	Group 1 (treated with illuminated TPP-doped NT) had a lesion area reduced from 12.5 to 8.1 cm^2^ (P < 0.01) while group 2 (untreated) had a lesion area reduced from 11.8 to 10.9 cm^2^ (P < 0.05). Group 1 showed a reduction in the levels of sphacelate, fibrin, an increase in granulation tissue and epithelialization on day 42. Patients reported that pain intensity had been reduced by 71% and 49% in group 1 and 2 respectively.	Clinical trial
8	Wound Rep Reg Medicine (plastic surgery)	2015 Switzerland	Poly-N-acetyl glucosamine nanofibers for negative-pressure wound therapies^(29)^	Nanofiber Commercial: NA	1- Poli-N-Acetyl glucosamine (sNAG) 2- NA	Pressure injuries	The application of sNAG nanofibers to the wound interface using negative pressure therapy (NPWT) was safe compared to using NPWT alone, leading to improved wound healing (16.4% versus 10.3%) due to greater stimulation of contraction rather than greater epithelialization.	Prospective randomized clinical trial.
9	J AM ACAD DERMATOL Nursing and Medicine	2012 USA	A randomized, investigator-blinded, controlled pilot study to evaluate the safety and efficacy of a poly-N-acetyl glucosamine derived membrane material in patients with venous leg ulcers^(30)^	Nanofiber Commercial: NA	1- Poli-N acetyl glucosamine (pGlcNAc) 2- NA	Venous ulcer	At 20 weeks, the proportion of patients with completely cured VUs was 45.0% (n = 9 out of 20), 86.4% (n = 19 out of 22) and 65.0% (n = 13 out of 20) for groups that received standard treatment plus pGlcNAc only once, every two weeks and every three weeks, respectively, versus 45.0% (n = 9 out of 20) for those who received standard treatment alone. The new pGlcNAc technology was well tolerated and safe.	Ensaio clínico randomizado, cego para o investigador e controlado
10	The Foot Medicine (endocrinology) and Pharmacy	2019 Egypt	The impact of topical phenytoin loaded nanostructured lipid carriers in diabetic foot ulceration^(26)^	Nanogel Commercial: NA	1- Nanostructured lipids (NLC) 2. Phenytoin (PHT)	Neuropathic diabetic foot ulcer (DFU)	The Nanogel (PHT-NLC Hydrogel) accelerates the healing process of DFU without any adverse effects when compared to the positive (PHT Hydrogel) and negative (Bank Hydrogel) control hydrogels. The reduction in area was 95.82 ± 2.22%, 47.10 ± 4.23% and 34.91 ± 28.33% for PHT-NLC, PHT and White respectively.	Randomized clinical trial
11	Current Nanomedicine Nanotechnology	2019 Mexico	Catalytic Nanomedicine. Cu/TiO2–SiO2 Nanoparticles as Treatment of Diabetic Foot Ulcer: A Case Report^(23)^	Nanogel Commercial: NA	1- Carboxymethylcellulose (CMC) and Polyacrylic acid (PAA) 2- Cu/TiO2-SiO2	Diabetic foot ulcer (DFU)	Cu/TiO2-SiO2 nanogel therapy improved re-epithelialization, significantly reduced its size and depth and accelerated the healing of a DFU. The successful outcome made it possible to avoid the amputation that was proposed for the patient.	Case study
12	Int J Low Extrem Wounds Medicine	2023 Egypt	Comparative Study Between Silver Nanoparticles Dressing (SilvrSTAT Gel) and Conventional Dressing in Diabetic Foot Ulcer Healing: A Prospective Randomized Study^(33)^	Nanogel Commercial: SilvrSTAT Gel^®^	1- Hydrogel 2-Silver nanoparticles	Non-ischemic diabetic foot ulcers (DFUs)	The healing rate of the SilvrSTAT Gel^®^ group was significantly higher than that of the control group (P < 0.0001). The rate of complete healing in the SilvrSTAT Gel^®^ group was achieved in 22 (55%), 29 (72.5%), 34 (85%) and 36 (90%) patients by the 6th, 8th, 10th and 12th weeks, respectively. In the control group: 20 (50%), 27 (67.5%) and 30 (75%) patients were completely cured by the 8th, 10th and 12th weeks, respectively.	Prospective, double-blind, randomized, controlled trial
13	Int Wound J	2023 Iran	Efficacy of topical atorvastatin-loaded emulgel and nano-emulgel 1% on post-laparotomy pain and wound healing: A randomized double-blind placebo-controlled clinical trial^(31)^	Nanoemulgel Commercial: NA	1- fat-soluble fatty acids and water-soluble polysorbates 2- Atorvastatin	Surgical wounds	On the Visual Analog Scale, healing accelerated by 57% and 89% and redness, edema and ecchymosis improved by 63% and 93% for the group treated with the emulgel and the group treated with the nanogel respectively in both scenarios.	Randomized, double-blind, controlled clinical trial
14	ADVANCES IN SKIN & WOUND CARE Medicine, Physics, Chemistry and Biology	2018 Sweden	Treatment of Nonhealing Ulcers with an Allograft/ Xenograft Substitute: A Case Series^(35)^	Nanomembrane Commercial: Eiratex^®^	1- NA 2- Biosynthetic cellulose	Chronic and acute venous ulcers	The use of Eiratex^®^ dressings reduced healing time (43 ± 6 days), the frequency of visits (5.7 ± 0.6) and dressing changes (1.7 ± 0.2) compared to the reports described in the literature for other materials. The use of Eiratex^®^ for wound healing can increase the quality of life of these patients and reduce costs for the healthcare system.	Series of cases
15	JOURNAL OF WOUND CARE Medicine	2018 Malaysia	Nano-colloidal silver and chitosan bioactive wound dressings in managing diabetic foot ulcers: case series^(34)^	Spray of Nanoparticles spray and Gel Biopolymer Commercial: SilvoSept Spray^®^ and ChitoHeal Gel^®^	1- NA 2- Nano-colloidal silver and chitosan	Diabetic foot ulcer (DFU)	Applications of nano-colloidal silver spray in conjunction with the bioactive gel chitosan as primary dressings in the management of DFU cases are safe and help to increase wound healing rates, reducing time and leading to significant cost savings in the hospital environment.	Series of cases (DFU)
16	SCIENCE DIRECT/BURNS Medicine (Dermatology)	2019 USA	A randomized comparative trial between Acticoat and SD-Ag in the treatment of residual burn wounds, including safety analysis^(24)^	Synthetic dressing Commercial: Acticoat^®^	1- Polyethylene and polyester mesh 2- Nanocrystalline silver	Burns	The nanocrystalline silver dressing (acticoat) showed a shorter healing time than silver sulphadiazine (12.42 ± 5.40) days vs (15.79 ± 5.60) days (p = 0.005) and a higher healing rate of 90.76 ± 14.45 vs 88.55 ± 15.64 (p = 0.508).	Multicenter randomized clinical trial
17	J Wound Ostomy Continence Nurs Nursing	2016 USA	Management of a Patient With Toxic Epidermal Necrolysis Using Silicone Transfer Foam Dressings and a Secondary Absorbent Dressing^(21)^	Synthetic dressing Commercial: NA	1- Silicon 2- Silver nanoparticles	Toxic epidermal necrolysis (NET)	The use of the dressing (foam) for topical treatment of a 77-year-old woman with NET affecting 90% of the SCA promoted epithelialization, reduced the trauma associated with frequent dressing changes and reduced pain during dressing changes. Epithelialization occurred within 12 days of starting this approach.	Case study
18	Journal of Wound Care Medicine Dermatology	2016 France	Quality of life in patients with leg ulcers: results from CHALLENGE, a double-blind randomised controlled trial^(25)^	Synthetic dressing Commercial: UrgoStart^®^	1- Lipid-Colloid (TLC) 2- Nano-oligosaccharide factor (NOSF)	Venous and mixed ulcers	The TLC-NOSF matrix dressing (UrgoStart) promotes faster healing of venous ulcers and mixed leg ulcers and significantly reduces pain/discomfort and anxiety compared to the TLC Matrix dressing (UrgoTull Absorb).	Double-blind randomized controlled clinical trial
19	IWJ WILEY Nursing	2021 Canada	A retrospective review of the use of a nanocrystalline silver dressing in the management of open chronic wounds in the community^(32)^	Synthetic dressing (NCS) Commercial: Acticoat^®^	1- Polyester 2- Nanocrystalline silver	Diabetic foot ulcer, venous ulcer, surgical ulcer and pressure injury	The average healing time for all types of wounds was reduced by more than half in patients treated with the NCS dressing (average 10.46 weeks) in contrast to the comparative treatment (average 25.49 weeks). The difference in the average labor cost of managing all types of wounds using the NCS dressing (CAN$1,251) proved to be significantly lower (p = 0.001) than the cost of wounds without the NCS dressing (CAN$6,488).	Retrospective non-experimental study
20	Wounds International Medicine	2021 China	Diabetic foot ulcer management with TLC-NOSF (Technology Lipido-colloid Nano oligosaccharide Factor) wound dressings^(36)^	Synthetic dressing Commercial: UrgoStart^®^	1- Lipid-colloid (TLC) 2- Nano-oligosaccharide (NOSF)	Diabetic foot ulcer (DFU)	The results, after the application of TLC-NOSF, represent a rapid improvement in the wound healing process through the reduction of the wound surface area.	Series of cases
21	Clinical Medicine Insights Medicine and Pharmacy	2019 Brazil	Evaluation of the Use of Compressive Stockings Impregnated with Hesperetin-Based Nanocapsules in the Healing of Venous Ulcers: A Case Report^(22)^	Compression stockings with Nanocapsules Commercial: NA	1- Textile fibers 2- Hesperetin nanocapsules	Venous ulcers	Macroscopically, the healing process was observable at 3 months of treatment. And 6 months later, a high percentage of retraction was observed in the area (92.8% and 93.1%) of the superficial lesions and (47.3%) in the area of the deepest lesion. The QoL and pain scores were 91.6 and 31.2/7 and 0, respectively. The reduction in venous diameters and melanin also indicates scar function.	Case study

Caption: 1 (Matrix), 2 (Nanoparticle or Bioactive incorporated), NA (Not applicable)

Source: Prepared by the authors.

Healing in diabetic foot ulcers has been described at around 95.8%^(26)^, 85%^(33)^, 81.8%^(27)^, 71%^(28)^ and a reduction of ±7 cm^(18)^, pressure sores at 16.4%^(29)^ and a reduction of ±15 cm^(20)^; leg ulcers a reduction of ±5 cm^(19)^ and a reduction of 43 days in treatment time^(35)^; venous ulcers healing was 92.8%^(22)^, 86.4%^(30)^; surgical wounds healing was 93%^(31)^ and burns 90.7%^(24)^.

As for the compounds associated with nanocomposites, a wide variety of products have been described: the aqueous extract of Pinus halepensis bark^(16)^, platelet-rich plasma^(17,28)^, mesenchymal stem cells^(28)^, venlafaxine and doxycycline^(18)^, tetraphenyl-porphyrin photosensitizer^(19)^, phenytoin^(26)^, Cu/TiO2-SiO2^(23)^, atorvastatin^(31)^, nanooligosaccharide factor (NOSF)^(25,36)^ and hespertine^(22)^.

## DISCUSSION

The technological advance inherent in the development of nanotechnology strengthens and disseminates in the academic field technological possibilities for resolving preponderant issues in the field of health^(37)^. The manufacture of nanocomposites for the health care of patients with skin lesions is being explored with this nanotechnological development, resulting in the supply of synthetic dressings with silver nanoparticles^(21,24,32,36)^, biomaterials with nanocrystalline silver^(27,34)^, nanofibers loaded with bioactive substances^(16,18,19,30,35)^, nanofibers loaded with growth factors^(17,28)^ or even synthetic dressings with growth factors^(25)^, hydrogels with growth factors^(26)^, nanogels^(23,33)^, nanoemulsions^(31)^ and the possibility of associating adjuvant therapies with nanocomposites^(22,29)^.

The transport of bioactive substances, growth factors and compounds such as silver through nanoparticles, nanogels, nanofibers and scaffolds adds to nanotechnology an efficient perspective for modulating the healing process and accelerating tissue repair^(17,18,28)^. The use of silver in nanocomposites has been explored repeatedly in studies aimed at studying the healing potential of nanostructures due to their added properties such as antimicrobial potential, antibiofilm and anti-inflammatory action^(21,24,32,33,36)^.

Nanocomposites with silver nanoparticles were the predominant object of study in the texts analyzed, with satisfactory results promoting accelerated tissue repair, control of microorganisms and modulation of the inflammatory process^(21,27,32-34,36)^. However, it should be used with caution due to its cytotoxicity to cells and tissues compared to classic ionic compounds^(27,34,38)^. The absorption of silver at the cellular level with a potential cytotoxic effect and the antimicrobial resistance of pathogens colonized in the wound bed as a result of prolonged use^(39,40)^ reinforce the need to control and ration the use of silver for a period of no more than four weeks^(41)^.

The use of silver nanocomposites has been described in epithelial lesions of different etiologies, with similar results in studies regarding the possibility of accelerating the healing process. In lesions resulting from burns, the silver nanocomposite promoted faster healing compared to the use of silver sulfadiazine^(24)^. In a case study of a patient with toxic epidermal necrolysis (TEN), the synthetic dressing with nanosilver promoted pain relief and rapid epithelialization^(21)^. A retrospective study of patients with venous lesions and pressure injuries showed that the use of a synthetic dressing with nano-silver reduced healing time by more than half, with a consequent reduction in hospital costs^(32,34)^. In diabetic foot lesions, the use of silver nanocomposites not only reduced the size of the lesion and healing time, but also promoted the control of microorganisms, since these patients tend to have a potential risk of infection, and reduced costs in the context of the hospital care provided^(27,33,34,36)^. However, the studies did not report the systemic use of antibiotic therapy, the performance of biopsies or tissue cultures, or any adverse effects from the use of silver in the patients included in the samples.

Because of their physical, chemical and optical properties, synthetic polymers are the preferred raw material for making nanofibers^(42,43)^. In the studies analyzed, they were preferentially chosen due to the excellent results resulting from their similarity to the extracellular matrix, thus contributing to the deposition of cells involved in the healing process and accelerating the regeneration of injured epithelial tissue^(16–20,25,28,29)^.

The possibility of incorporating bioactive compounds or components gives nanocomposites biocompatibility characteristics and also potentials such as anti-inflammatory, antioxidant and antimicrobial activity, which make it possible to accelerate the healing of skin lesions^(16,25,27,34,35)^. Evidence strongly supports the fact that nanotechnology makes it possible to incorporate bioactive substances such as cellulose, chitosan and curcumin, giving the nanocomposite not only organic and cellular biocompatibility, but also additional properties that enhance its results and possible applications^(39,44,45)^.

Studies with the addition of cellulose^(16,18,35)^, collagen^(20)^ and chitosan^(34)^ to nanostructures corroborate the evidence with similar results in promoting healing in less time^(16,18,20,34,35)^, with a reduction in changes and costs^(34,35)^, improving quality of life^(20)^. The biocompatibility of the nanocomposite led to a reduction in peripheral neuropathy^(18)^ and a reduction in erythema and pruritus^(16)^.

Not only the incorporation of natural compounds, but also the incorporation of growth factors as a strategy to enhance wound healing can be seen to be quite effective^(25,26,28,36)^. Although they can contribute, none of the studies have made a comparative analysis of the isolated effect of the components carried by the nanocomposites.

The complexity of the healing process of skin lesions, as it involves numerous cellular structures, such as growth factors and cytokines, makes it imperative to develop technology and materials capable of succeeding and modulating the action of these structures in the lesion bed^(37,46)^. Significant advances in nanotechnology in the last decade have made it possible to incorporate growth factors into nanofibers, nanogels and scaffolds with a positive and evident effect on reducing tissue repair time^(17,25,26,28,33)^.

It was possible to observe through this review that the use of nanocomposites is not only emerging as an individual therapy for the healing process, but also in association with advanced adjuvant treatments such as compressive therapy and negative pressure therapy, with congruent outcomes accelerating the healing process^(19,22)^. Nanotechnology is emerging as a therapeutic possibility for skin lesions, either individually or in combination, and its concomitant use with therapies described in various guidelines on the subject is referenced as safe, beneficial and effective, with results maximized by combining adjuvant therapies with nanotechnology^(1,11,47)^.

In isolation or in association with advanced adjuvant treatments, the use of nanocomposites has shown significant efficacy in reducing the healing time of skin lesions in chronic and morbid patients, contributing to an effective reduction in the costs incurred by healthcare institutions in providing care^(32,34,35)^. The chronicity of skin lesions results in long periods of hospitalization with prolonged occupation of hospital beds and outpatient vacancies, and the availability of therapeutic measures or effective materials is necessary to reduce the costs resulting from delayed healing in patients^(32,34,35)^. With the results presented, nanotechnology has emerged as an effective therapeutic measure in the production of nanomaterials capable of reducing healing time and consequently reducing healthcare costs for patients with chronic injuries^(10,11,38)^.

The progress of nanotechnology as a therapeutic for skin lesions depends on the development of biocompatible nanomaterials that favor the wound healing process and this involves understanding the interaction of nanomaterial components with the lesion bed and the factors involved in the complex healing process. It is imperative that health professionals involved in caring for people with skin lesions take ownership of nanotechnology. The positive results make nanofibers, scaffolds, nanogels and nanomaterials associated with biomaterials an efficient technology to implement.

This review identified restrictions on the use of nanomaterials by professionals directly involved in caring for skin lesions, such as nurses, with a limited number of studies conducted by these professionals. In addition, few clinical research studies were identified involving large numbers of patients and the limited use of nanomaterial manufacturing techniques in the areas of tissue engineering.

The limitations of this study include the fact that other eligible studies may not have been included because they were not indexed in the databases selected for this review and were not retrieved by the gray literature search, despite the fact that a broad and highly sensitive search was carried out in the sources investigated. Furthermore, no methods or instruments were used to assess the quality of the studies included.

## CONCLUSION

This study showed that the use of nanofibers, nanogels, nanoemulsions and nanomembranes in isolation or associated with adjuvant therapies such as negative pressure therapy and compression therapy is a therapeutic possibility for the treatment of skin lesions, with nanocomposites being effective in speeding up the healing process and reducing healthcare costs. The possibility of adding bioactive substances increases additional properties to the nanocomposites to modulate the tissue regeneration process.

The results of this scoping review still show insufficient results for the application of nanomaterials in the field of human research, with regard to their use in skin lesions, since the number of phase III clinical trials found is still low in the literature consulted.

It can be seen that the field of study is little explored in the national literature, and is more explored internationally, reflecting a large gap to be filled with future research, in view of the emerging need for research into low-cost synthetic nanocomposites and/or sustainable biomaterials for the treatment of skin lesions.
